# Circulating extracellular vesicles are monitoring biomarkers of anti-PD1 response and enhancer of tumor progression and immunosuppression in metastatic melanoma

**DOI:** 10.1186/s13046-023-02808-9

**Published:** 2023-09-28

**Authors:** Simona Serratì, Roberta Di Fonte, Letizia Porcelli, Simona De Summa, Ivana De Risi, Livia Fucci, Eustachio Ruggieri, Tommaso Maria Marvulli, Sabino Strippoli, Rossella Fasano, Tania Rafaschieri, Gabriella Guida, Michele Guida, Amalia Azzariti

**Affiliations:** 1 IRCCS Istituto Tumori Giovanni Paolo II, V.Le O. Flacco, 65, 70124 Bari, Italy; 2 Department of Basic Medical Sciences Neurosciences and Sense Organs, University of Bari, Piazza G. Cesare, 11, 70124 Bari, Italy

**Keywords:** Extracellular vesicles, Metastatic melanoma, Predictor of anti-PD1 response, Anti-PD1 resistance

## Abstract

**Background:**

Clinical drawback in checkpoint inhibitors immunotherapy (ICI) of metastatic melanoma (MM) is monitoring clinical benefit. Soluble forms of PD1(sPD1) and PD-L1(sPD-L1) and extracellular vesicles (EVs) expressing PD1 and PD-L1 have recently emerged as predictive biomarkers of response. As factors released in the blood, EVs and soluble forms could be relevant in monitoring treatment efficacy and adaptive resistance to ICI.

**Methods:**

We used pre-therapy plasma samples of 110 MM patients and longitudinal samples of 46 patients. Elisa assay and flow cytometry (FCM) were used to measure sPD-L1 and sPD1 concentrations and the percentage of PD1^+^ EVs and PD-L1^+^ EVs, released from tumor and immune cells in patients subsets. Transwell assays were conducted to investigate the impact of EVs of each patient subset on MM cells invasion and interaction between tumor cells and macrophages or dendritic cells. Viability assays were performed to assess EVs effect on MM cells and organoids sensitivity to anti-PD1. FCM was used to investigate immunosuppressive markers in EVs and immune cells.

**Results:**

The concentrations of sPD1 and sPD-L1 in pre-treatment and longitudinal samples did not correlate with anti-PD1 response, instead only tumor-derived PD1^+^ EVs decreased in long responders while increased during disease progression in responders. Notably, we observed reduction of T cell derived EVs expressing LAG3^+^ and PD1^+^ in long responders and their increase in responders experiencing progression. By investigating the impact of EVs on disease progression, we found that those isolated from non-responders and from patients with progression disease accelerated tumor cells invasiveness and migration towards macrophages, while EVs of long responders reduced the metastatic potential of MM cells and neo-angiogenesis. Additionally, the EVs of non-responders and of progression disease patients subset reduced the sensitivity of MM cells and organoids of responder to anti-PD1 and the recruitment of dendritic cells, while the EVs of progression disease subset skewed macrophages to express higher level of PDL-1.

**Conclusion:**

Collectively, we suggest that the detection of tumor-derived PD1 + EVs may represent a useful tool for monitoring the response to anti-PD1 and a role for EVs shed by tumor and immune cells in promoting tumor progression and immune dysfunction.

**Graphical Abstract:**

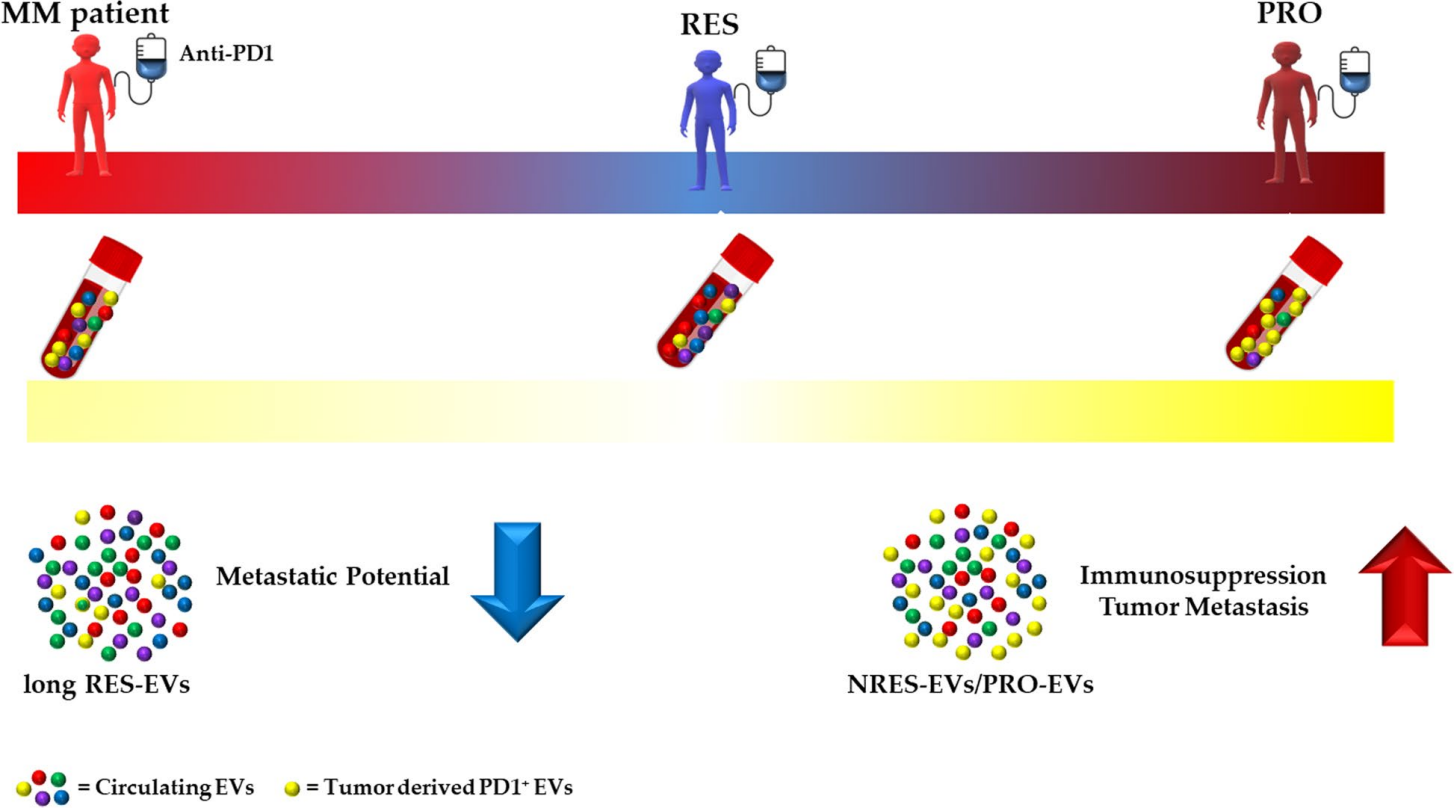

**Supplementary Information:**

The online version contains supplementary material available at 10.1186/s13046-023-02808-9.

## Background

Since 2011, continued advance in melanoma treatment strategies improved significantly the survival of patients with stage IV disease; the advent of immune checkpoint inhibitors (ICI) targeting programmed cell death protein 1 (PD1) as monotherapy or in combination with cytotoxic T-lymphocyte-associated protein 4 (CTLA-4) inhibitors ushered in a Copernican revolution in the treatment of metastatic melanoma that rapidly spread to a large spectrum of tumors [1, 2]. Nevertheless, clinicians still observe a lack of therapy response for about the 50% of these patients because they have primary or develop acquired resistance to these immunotherapy approaches [3–5].

To raise this bar, the whole scientific community aims to discover suitable markers that can allow the selection of patients who could respond to anti-PD1 as well as the monitoring of the immunotherapy response for the early detection of acquired resistance. The latter would lead to the consequent suspension of treatment, reducing the toxicity to patients and the economic impact on the national health system. This research field has shifted the identification of immune-related biomarkers from tissue to liquid biopsies, therefore with a negligible impact on patient compliance. Among the possible biomarkers, a strategic role has been attributed to the circulating extracellular vesicles (EVs) [6].

We have recently demonstrated that circulating PD1^+^ EVs are driver of innate resistance to anti-PD1, by sequestering the PD-1 blocking antibodies, and highlighted that the determination of the percentage of three circulating EV subpopulations (PD1^+^ EVs from T cells and B cells and PD-L1^+^ EVs from melanoma) by liquid biopsy is a promising tool to select MM patients for treatment with Nivolumab or Pembrolizumab [7, 8].

In literature, the soluble forms of the immune checkpoints, sPD1 and sPD-L1, have also been the topic of intense research and studies to clarify their role as biomarkers of response to anti-PD1. Their levels were the highest in the plasma of MM patients during anti-PD1 treatment [9, 10]. The analysis of sPD1 and sPD-L1 is technically easier to perform, since it does not provide for the purification of circulating EVs as we did previously [7, 8]. Therefore, we performed a basal and longitudinal assessment of the plasma levels of sPD1 and sPD-L1 in order to investigate their role as monitoring biomarkers of the response to ICI treatment.

Likewise we tested as biomarkers for monitoring the response to ICI the circulating EVs isolated from the same serial longitudinal plasma samples described before.

The neverending story on the role of circulating EVs in the response and monitoring of the response to ICI in MM patients has been enriched with the investigation of possible correlation between circulating EVs in plasma of patients and the developing of acquired anti-PD1 resistance and in modulating the behaviour of both tumor and immune cells, with a direct impact on the metastatic potential of tumor cells and on the regulation of immune tolerance in cells strictly involved in the response to anti-PD1, such as T lymphocytes (T cells), dendritic cells (DCs) and macrophages.

## Methods

### Aim

We sought to investigate the relevance of circulating EVs in the monitoring of clinical response of MM patients treated with anti-PD1 and to elucidate possible mechanisms underlying pro-tumors and immunosuppressive activity of EVs.

### Study design

The study is composed of 3 main sections: 1) the investigation of the soluble forms of PD1 and PD-L1 as monitoring biomarkers for the response to ICI; 2) the evaluation of selective populations of circulating EVs as monitoring biomarkers for the response to ICI and 3) the characterization of how circulating EVs support tumor progression and immunosuppression.

### Patients and study design

The study on the circulating EVs was previously approved by the Ethics Committee of the IRCCS Istituto Tumori Giovanni Paolo II (Prot. 590/CE) and written informed consent was obtained from all the patients enrolled in the study. Blood samples were collected from 110 MM patients treated with checkpoint inhibitors at IRCCS Istituto Tumori Giovanni Paolo II from January 2017 to December 2021 whose characteristics are reported in Table 1.

**Table 1:** Main characteristics of patients at immunotherapy (*n* = 110)

Characteristic	n (%)
Age at metastasis, years, median [range]	60 [31–92]
Sex male	60 (55)
Basal LDH^a^	
Normal	60 (54)
Increased	46 (42)
N of metastatic sites^b^	169 (62.1)
< 3	73 (66)
≥ 3	36 (33)
Site of melanoma	
Cutaneous	87 (79)
Mucosal	3 (3)
Ocular	3 (5)
unknown	14 (13)
Prior Adjuvant therapy	6 (5)
Prior therapy for metastatic disease	47 (43)
ECOG PS^c^	
0	57 (52)
1	42 (38)
2	9 (8)
Stage at metastatic disease	
M1a	30 (27)
M1b	19 (17)
M1c	45 (41)
M1d	16 (15)
Therapy	
Anti-PD-1	106 (96)
Anti-PD-1 plus Anti-CTLA-4	4 (4)

^a^4 patients had missing value

^b^ 1 patient had missing value

^c^3 patient had missing value

Plasma samples were divided into two groups consisting of i) pre-therapy (basal) samples from 110 MM patients: 39 responders (RES) and 71 non-responders (NRES) and ii) serial samples from 46 of these patients treated with anti-PD1: 25 NRES, 15 long RES and 6 MM patients who initially responded to ICI and then progressed (RES > PRO). Baseline sampling was available for all patients and sampling at the time of clinical re-evaluation (median value: 4 months) was available for NRES when the patient showed innate resistance to anti-PD1. For long RES, in addition to the baseline sample, samples were taken at the first re-evaluation (median value: 4 months) and after 21.5 months (median value) during which they showed partial or complete response. For RES > PRO, the blood samples were collected pre-therapy, at the best response (median value: 4 months) and at the time of progression (median value: 32.5 months). Samples were taken before starting immunotherapy for all patients and for the group enclosed in the longitudinal study at two/three times as reported in Table 2.

**Table 2:** Timing of sample collection

Basal samples		Longitudinal study				
**Blood sample**	**Pre-therapy (n.)**	**Blood sample**	**Pre-therapy (n.)**	**first re-assessment (n.)**	**Second re-assessment** **(n.)**	**at progression** **(n.)**
NRES	X (71)	NRES	X (25)	X (25)		
RES	X (39)	long RES	X (15)	X (15)	X (15)	
		RES > PRO	X (6)	X (6)		X (6)
**TOTAL**	**110**	**46**				

### Establishment of a primary culture of Metastatic Melanoma cells (MGS)

The study for in vitro/ex vivo MM model preparation was previously approved by the Ethics Committee of the IRCCS Istituto Tumori Giovanni Paolo II (Prot. 737/CE) and written informed consent was obtained from all the patients enrolled in the study. The surgical specimen was obtained from an adult patient with metastatic melanoma enrolled in our Institutional Ethical Committee‐approved protocol (Prot. 737/CE) and conducted in accordance with the international standards of good clinical practice. Informed consent was previously signed by the patient. The preparation of biopsy-derived tumor cells, named MGS, was carried out from the surgical specimen as previously described [11]. MGS cells, BRAF wt, were characterized for the expression of the two conventional melanoma markers, PMEL and S100B in immunofluorescence (IF) as described in [12].

### Cell lines and culture conditions

In the current study, two melanoma cell lines BRAF wild type (MGS and LND-1) and two BRAF mutated (BRAFV600) (Hmel-1, Hmel-9) were utilized [13]. Hmel-1, Hmel-9 and MGS were extracted from skin metastases obtained from human sporadic melanoma biopsy specimens after the informed consent of patients. Cells were grown in high-glucose Dulbecco's modified Eagle's medium (DMEM) supplemented with 10% (v/v) fetal bovine serum (FBS), 1% (v/v) L-glutamine, 1% (v/v) penicillin/streptomycin. H-MVECs (human dermal microvascular endothelial cells) were purchased from Lonza (Basel, Switzerland). The EGMTM-2 Endothelial Growth Medium-2 BulletKitTM from Lonza, Switzerland, was used for H-MVEC cell culture. The human monocytic leukemia cell line THP-1 (ATCC® TIB202™) was generously gifted by Dr. MC Vegliante (Hematology and Cell Therapy Unit, IRCCS Istituto Tumori G. Paolo II, Bari) and was cultured in ATCC-formulated RPMI-1640 medium, supplemented with 10% (v/v) FBS, 1% (v/v) penicillin/streptomycin and 2-mercaptoethanol to a final concentration of 0.05 mM. To differentiate THP-1 into macrophages the cells were treated with 320 nM phorbol-12-myristate-13-acetate (PMA—Sigma Aldrich, USA) for 6 h. To generate M1 or M2-polarized THP-1 macrophages, THP-1 cells were treated with 320 nM PMA for 6 h and then added 100 ng/mL LPS (Miltenyi Biotec, Germany) and 20 ng/mL IFN-g (Miltenyi Biotec, Germany) or 20 ng/mL IL-4 (Miltenyi Biotec, Germany) and IL-13 (Miltenyi Biotec, Germany) for another 18 h, respectively, as described in [14]. DCs were generated after incubating THP-1 cells with 20 ng/mL of IL-4 and 20 ng/mL of PMA for 4 days, followed by 1 μg/mL LPS for 48 h, as described in [15]. All cells were cultured at 37 °C in a humidified atmosphere at 5% CO_2_.

### Generation of patient-derived organoids (PDOs)

The study for in vitro/ex vivo MM model preparation was previously approved by the Ethics Committee of the IRCCS Istituto Tumori Giovanni Paolo II (Prot. 737/CE) and written informed consent was obtained from all the patients enrolled in the study. PDOs were obtained from the surgical specimen of a RES enrolled in our Institutional Ethical Committee‐approved protocol (Prot. 737/CE) and conducted in accordance with the international standards of good clinical practice. Informed consent was signed by the patient. The tumor tissue was processed as previously described [16] and cultured in Advanced DMEM F12 (GibcoTM, USA) supplemented with 10% (v/v) FBS (GibcoTM, USA), B27 (GibcoTM, USA), R-spondin3 (R&D System, USA), Glutamax (GibcoTM, USA), Noggin (PeproTech, USA) and antibiotics and maintained in ultra-low attachment tissue culture plates.

### Blood and plasma sample collection

Peripheral blood were collected in sodium citrate tubes and plasma isolated as described in [8].

### Determination of PD1 and PD-L1 in plasma patients

Both sPD1 and sPD-L1 were determined in plasma patients by the Human PDCD1/CD279/PD1 ELISA Kit (LSBio, LifeSpan BioSciences, Inc., Washington, US) and the Human PD-L1 SimpleStep ELISA Kit (28–8) (Abcam, Cambridge, UK) following the manufactured instructions.

### PBMC isolation

PBMCs were separated from other components of the peripheral blood via density gradient centrifugation using Ficoll-Hypaque. 5–8 ml of blood were processed as previously described [8] and the isolated cells were cryopreserved at -195 °C.

### EVs isolation

The EVs were isolated from 5 ml of fresh plasma. The samples were centrifuged for 15 min at 2600xg [17] and the supernatant was diluted 1:1 in PBS and filtered with 200-nm pore size filters. The resulting plasma was processed according to the MISEV18 line guides [18], by ultracentrifugation [19]: one step at 10000xg for 30 min and then twice at 100000xg for 70 min. The EVs were then pooled in aliquots and stored at—80 °C [18, 20].

### Nanoparticle tracking analysis (NTA)

EVs were analyzed with the NanoSight NS300 (Malvern Panalytical) following the manufacturer’s instructions (NanoSight NS300 User Manual, MAN0541-02-EN, 2018) and according to MISEV18 line guides [18]. A volume of 1–2 µL of each EV sample were diluted 1:1000 and the particles was measured at constant syringe flow (flow rate = 50) using the sCMOS camera as previously described [7].

### EV characterization by flow cytometry (FCM)

The FCM instrument preparation and setup was performed as described in [8]. The EVs samples were incubated with Super Bright Complete Staining Buffer (eBiosciences, Invitrogen) according with the manufacturer’s instructions. Then the EVs were labelled with the anti-human antibodies and analyzed using an Attune TMNxT Acoustic Focusing Cytometer (ThermoFisher) as described [8]. To distinguish the cell origin of EVs we used: anti-human CD146 for melanoma calls, anti-human CD1a for DCs, anti-human CD8 for T cells, anti-human CD14 for monocytes and anti-human CD19 for B cells.

### Primary labelled antibodies

The panel of the primary labelled antibodies for EVs characterization were obtained by eBiosciences (Thermo Fisher Scientific, Waltham, MA USA): anti-human-CD9 (FITC, Clone: eBioSN4, SN4 C3-3A2) (0.125 µg/test), anti-human CD63 (PE-CYN7, Clone: H5C6) (0.5 µg/test), anti-human CD81 (APC, Clone: 1D6) (1 µg/test), anti-human CD146 (PE, Clone: P1H12) (0.125 µg/test), anti-human CD1a (eFluor-450, Clone: H149) (0.5 µg/test), anti-human CD8 (PE-CYN5, Clone: RPA-T8) (0.25 µg/test), anti-human CD14 (PE-EF610, Clone: 61D3) (0.25 µg/test), anti-human CD19 (EF506, Clone: HIB19) (0.5 µg/test), anti-human CD274 (PD-L1, B7-H1) (Alexa Fluor® 700, Clone: MIH1) (1 µg/test), anti-human CD279 (PD1) (Super Bright 600, Clone:eBioJ105, J105) (0.5 µg/test), anti-human CD223 (LAG-3) (Super Bright 702, Clone 3DS223H) (0.25 µg/test), anti-human CD87 (uPAR) (PerCP-eFluor 710, Clone VIM5) (0.25 µg/test). For the evaluation of PD-L1 expression in macrophages we used: anti-human CD68 (PE-eFluor 610, clone eBioY1/82A) (0.25 µg/test) and anti-human PD-L1 (APC-eFluor 780, Clone M1H1) (0.5 µg/test), purchased from eBiosciences (Thermo Fisher Scientific, Waltham, MA USA). For the characterization of the primary cell line MGS, anti-human S100B (Novus Biological, Bio-Techne SRL, Milano, Italy) and anti-human PMEL (OriGene Technologies, Inc., Rockville, MD, USA) and the fluorescent Alexa dye-labeled secondary antibodies (Alexa Fluor 488 and Alexa Fluor 568, Invitrogen, ThermoFisher Scientific, Waltham, MA USA) were utilized.

### Invasion assay in Boyden chambers

Invasion ability through matrigel-coated porous filters of MM cell lines was evaluated either in absence or presence of the pooled EVs from 3 NRES, 3 long RES and 3 RES > PRO, as previously described [21]. Briefly, 3 × 10^4^ cells were plated onto the upper side of the chamber, while 2 × 10^7^ EVs were placed in the lower compartment. The invasion was allowed to occur for 6 h for Hmel-1 and Hmel-9 or 18 h for LND-1 and MGS respectively. After the cell incubation at 37 °C, the migrated cells were stained by Diff-Quick (Polysciences, Inc., Polysciences Europe GmbH, Eppelheim, Germany) and counted by light microscopy (40X magnification). Experiments were performed in triplicate. Cell invasion was expressed as mean ± SD of the number of total cells counted/filter.

### Capillary morphogenesis

In vitro capillary morphogenesis was performed to evaluate the ability of H-MVECs to organize in interconnecting tubular structures on Matrigel in presence of EVs. 50 µL of Matrigel (10–12 mg/mL) was pipetted into 0.64 cm (diameter) tissue culture wells and polymerized for 30 min to 1 h at 37 °C, as described in [22]. H-MVECs were plated (3 × 10^4^/mL) in EGM-2 (endothelial growth medium, EGM™-2MV BulletKit™ Lonza, Switzerland) with 2% of exosome-depleted FBS (FBS South America, exosome depleted, Bio West, France). The assay was performed in the presence of the pooled EVs (2 × 10^7^) from 3 NRES, 3 long RES and 3 RES > PRO. Six to nine photographic fields from three plates were scanned for each point.

### Cytotoxicity assays

To assess the immune-mediated cytotoxicity of Pembrolizumab (Selleckchem, USA), in presence or absence of circulating EVs, we tested the drug in 2D culture of BRAF wt LND-1 cell line and in RES-PDOs, by adding activated autologous PBMCs. LND-1 cells were seeded 5,000 cells/well and, after 24 h, treated with 20 µg/mL Pembrolizumab. PBMCs from a RES were previously activated with immunocult human aCD3/aCD28 T cell activator (STEMCELL Technologies, Canada) for 72 h and then added to the wells in a ratio 1:5 (tumor cells-PMBCs). Additionally, 10^7^ EVs from NRES, long RES and RES > PRO were added to the treated samples. After 72 h treatment, PBMCs were removed from the wells and tumor cells cytotoxicity was evaluated by MTT assay, as previously described [23]. For the cytotoxicity assay in PDO model we used the same treatment conditions as in the 2D model except for EVs of which 10^8^ EVs were added because organoids contain a high amount of tumor, stromal and immune cells (about 100,000 cells). After 72 h treatment we assessed the viability by Cell Titer-Glo 3D Cell Viability Assay (Promega, USA), according to the manufacturer’s instruction and as described in [16].

### Transwell Migration assay

The transwell migration assay was used to evaluate the ability of the tumor or immune cells to directionally respond to circulating EVs stimuli, as previously described in [24]. Briefly, 1.2 × 10^5^ cells (tumor cells or DCs or machrophages) were seeded on top of the 8 µm-pores filter membrane in a 24-well transwell insert and allowed to settle down at 37 °C and 5% CO_2_. In the lower well we seeded 2 × 10^5^ cells (tumor cells or macrophages or none) and after attachment, we added 10^8^ EVs from NRES, long RES and RES > PRO for 6 h. Afterwards, the insert was placed in the well containing the pre-incubated cells with the circulating EVs and the plate was incubated for 18 h. The transwell insert was withdrawn from the plate and the media and remaining cells that have not migrated were removed by a cotton-tipped applicator. Then, migrated cells were fixed by 70% ethanol, stained with 0.2% crystal violet and counted by using an OLYMPUS CKX41 microscope (OLYMPUS, Tokyo, Japan). Migration rate was expressed as mean ± SD of the number of migrated cells counted/filter.

### PD-L1 evaluation by FCM

The PD-L1 expression on PMA differentiated-THP-1 macrophages (M0) after a co-incubation with circulating EVs from NRES, long RES and RES > PRO was assessed by FCM. The cells were seeded at a density of 3 × 10^5^/well in 6-well plates and incubated at 37 °C and 5% CO_2_ to allow attachment. Then, 2 × 10^8^ circulating EVs were added in each well and the plates were incubated for 18 h. Afterwards, the cells were harvested, washed twice, resuspended in ice-cold PBS without Ca^2+^ and Mg^2+^, and stained with anti-human PD-L1 (APC-eFluor 780, Clone M1H1) for 30 min at 2–8 °C in the dark. After staining, cells were washed and analyzed using an Attune NxT Acoustic Focusing Cytometer (Thermo Fisher Scientific, Waltham, MA, USA) and Attune NxT Analysis Software (Thermo Fisher Scientific, Waltham, MA, USA).

### Cytokines and chemokines determination

The release of cytokines and chemokines from M0 after the addition of circulating EVs of various origins was measured by using a Bio-Plex Pro Human Cytokine 27-plex assay (Biorad Laboratories, USA). The cells were seeded and co-incubated with circulating EVs from NRES, long RES and RES > PRO, as described above. After 18 h co-incubation, the supernatant was collect and centrifuged to discard the remaining cells. The assay was carried out according to the manufacturer’s instructions and analysed by Bio-Plex 200 system and Bio-Plex Manager software v. 6.1.1 (Biorad Laboratories, USA).

### Statistics

Statistical significance was calculated using two-tailed t-tests, Mann–Whitney U tests and two-tailed ANOVA using GraphPad Prism V.5.0 software (GraphPad Software, San Diego, California, USA). Survival analyses and test for equality of proportions has been performed through R v.3.6.3 environment. Statistical significance: *p < 0.05, **p < 0.01, and ***p < 0.001.

## Results

### Clinical outcomes

The clinical characteristics of patients (age, sex, comorbidities), of primary disease (melanoma type, stage at diagnosis, anatomical site of primary melanoma, Breslow, mitosis, etc.) and of metastatic disease (tumor burden, metastatic sites, LDH, therapies performed) were summarised in Table 1. The median age of the population was 60 years (range 31–92), of which 55% were male. Regarding the origin of melanoma, 79% were cutaneous and 13% of unknown origin. At metastatic disease, the 66% of patients had 1 or 2 metastatic sites. Stage M1c was the most represented (41%), while LDH was upper the normal value in 42% of patients. The ECOG PS was 0 or 1 in the 90% of patients. Forty-three % of patients underwent at least 1 line of therapy prior to immunotherapy (40 patients: BRAFi/ MEKi, 5 patients: ipilimumab, 1 patient: both ipilimumab and chemotherapy, and 1 patient: both ipilimumab and BRAFi/ MEKi). Almost all patients received anti PD-1 therapy alone (96%), while 4% were treated with the combination anti-PD-1 plus anti-CTLA-4.

### Can circulating soluble forms of PD1 and PD-L1 predict response to anti-PD1 and/or allow monitoring of response to this therapy?

The first step of the study in liquid biopsy consisted of determining the plasma levels of sPD1 and sPD-L1 in a cohort of MM patients who subsequently underwent immunotherapy with anti-PD1.

**Fig. 1:**
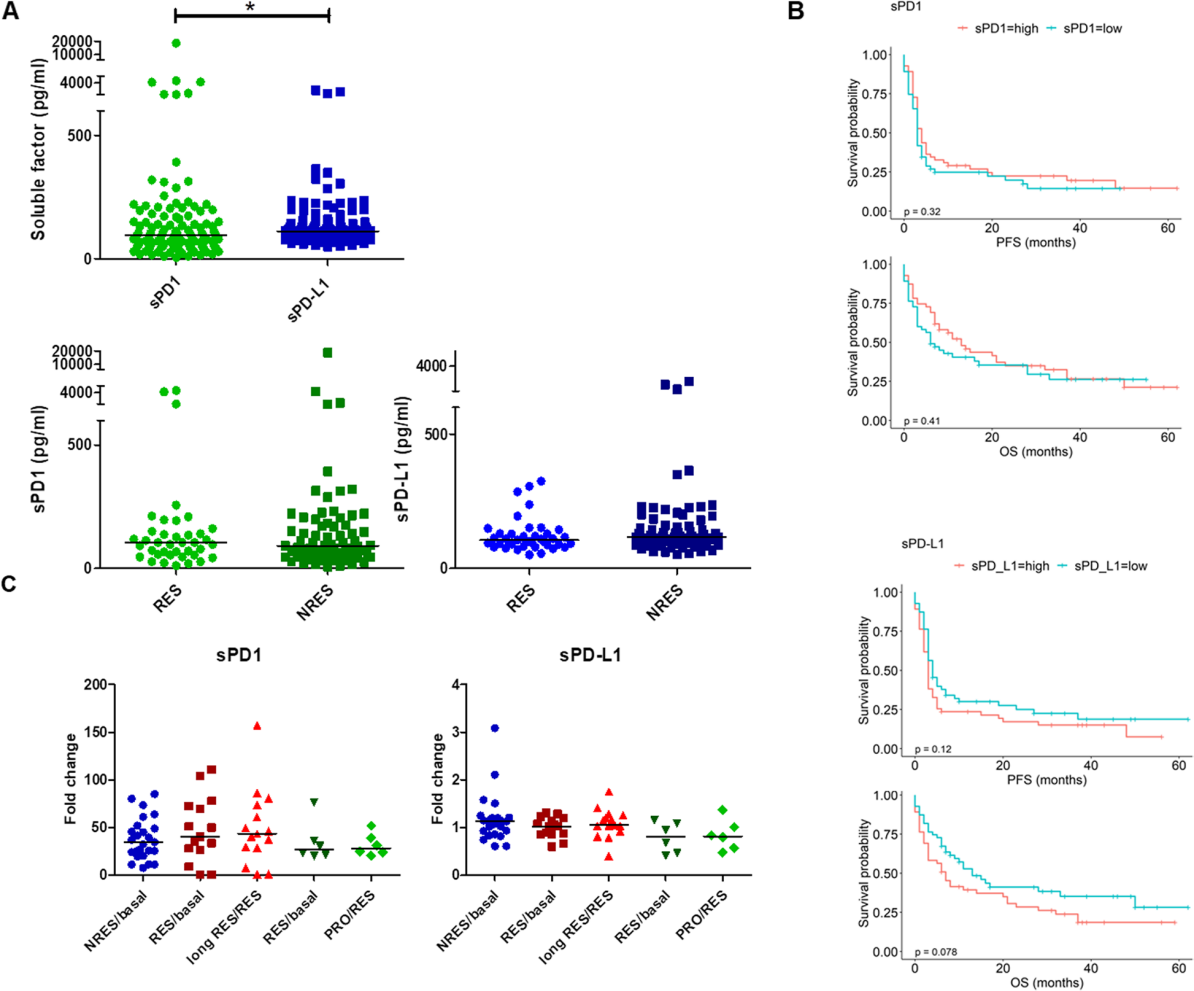
sPD1 and sPD-L1 levels in plasma of MM patients underwent immunotherapy with ICI. **A** Violin plots with median showing sPD1 and sPD-L1 plasma levels (pg/mL) in MM patients (RES and NRES) measured by ELISA assay (*p < 0.05). **B** Kaplan–Meier survival curve of MM patients with high or low sPD1 and sPD-L1 level as respect to PFS and OS. **C** Scatter plots with median showing sPD1 and sPD-L1 plasma levels fold change of the first-response/basal and of the second-response/first-response or of the progression/response of NRES, long RES and RES > PRO MM patients

The analysis reported in Fig. 1A showed that the basal level of sPD-L1 in MM patients was statistically higher than that of sPD1 with p = 0.0150; analysing the two soluble checkpoints in NRES e RES, the median values of sPD1 were 126.24 pg/ml and 104.98 pg/ml, and for sPD-L1 were 146.57 pg/ml and 106.53 pg/ml, respectively. In RES there were not differences between sPD1 and sPD-L1 concentration level, conversely in NRES sPD-L1 (146.57 pg/ml) concentration was higher than sPD1 (126.24 pg/ml) with *p* = 0.0106. Moreover, there were no statistically significant differences between patients with low and high levels of sPD1 and sPD-L1 in terms of the clinical outcome (PFS and OS), as shown in the Kaplan–Meier survival curves (Fig. 1B). Thus, the soluble forms of PD1 and PD-L1 can’t be considering promising biomarker for the selection of MM patients to treat with ICI.

Indeed, the analysis of sPD1 and sPD-L1 modulation in the longitudinal study evidenced that the anti-PD1 induced a very strong increase in soluble levels of PD1 which remained constant over time regardless of the clinical response. For sPD-L1 there were lower and no statistically significant changes as a function of therapy time (Fig. S1). Unfortunately, the analysis reported in Fig. 1C highlighted no statistically significant differences in the variation of their plasma levels in the samples of both long RES and of RES > PRO, if we compare the ratio of the concentrations evaluated at the time of first response over the basal one (first response/basal) and the ratio of the concentrations evaluated at the time of the second response over the first response (second response/ first response). Thus, both sPD1 and sPD-L1 can’t be use for predicting the response to anti-PD1 or for the monitoring of ICI effectiveness suggesting us to move on to the characterization of circulating EVs for searching biomarkers of ICI monitoring.

### Can circulating PD1^+^EVs, PD-L1^+^EVs and/or uPAR^+^EVs be used to monitor the response to the anti-PD1?

The investigation of changes in the level of circulating EVs, positive for PD1, PD-L1 and uPAR as a function of therapy time have been carried out by using liquid biopsy of the same series of MM patients included in the previously described study, in order to explore their role as monitoring factors of anti-PD1 response as well as biomarkers useful for the selection of MM patients to undergo immunotherapy [8]. These analyzes were performed on EVs from: 25 NRES, 23 from the old cohort [7, 8] and 2 subsequently enrolled, 15 long RES and 6 RES > PRO, all from the previous cohort [7, 8].

The EVs were isolated and characterised following the MIVEV2018 guidelines [18] and as detailed in our previous papers [7, 8]. As representative of the characterization approach applied, the NTA histogram of circulating EVs isolated from the plasma of a MM patient, enrolled in the study, with confidence interval in red reporting the concentration and specific particle size of EVs, is shown in Fig. S2A together with the FCM analysis of the double positivity for CD9/CD63, CD9/CD81 and CD63/CD81, that we utilised to perform the identification of circulating EVs [8] (Fig. S2B). The percentage of the circulating EVs coming from melanoma cells, T cells, B cells, monocytes and dendritic cells of all pre-therapy samples, included in the longitudinal study, are reported in Fig. S2C, showing that the highest and the lowest ones were those from monocytes and B cells, respectively. Even when the samples were analysed by stratifying them into the three subpopulations, NRES, long RES and RES > PRO, the trend did not change (Fig. S2C). In the longitudinal study, the ratio of the circulating EVs of different origin isolated from the plasma of the three patient populations, NRES, long RES and RES > PRO, were statistically over 1 only for EVs from B cells in long RES, indicating their increase after immunotherapy (Fig. S2D). However, an increasing trend of the release of EVs from T and B cells after administration of anti-PD1 was evident in EVs from NRES and in all subsets of EVs, with the exception of those isolated from melanoma cells in long RES and RES > PRO (Fig. S2D).

**Fig. 2:**
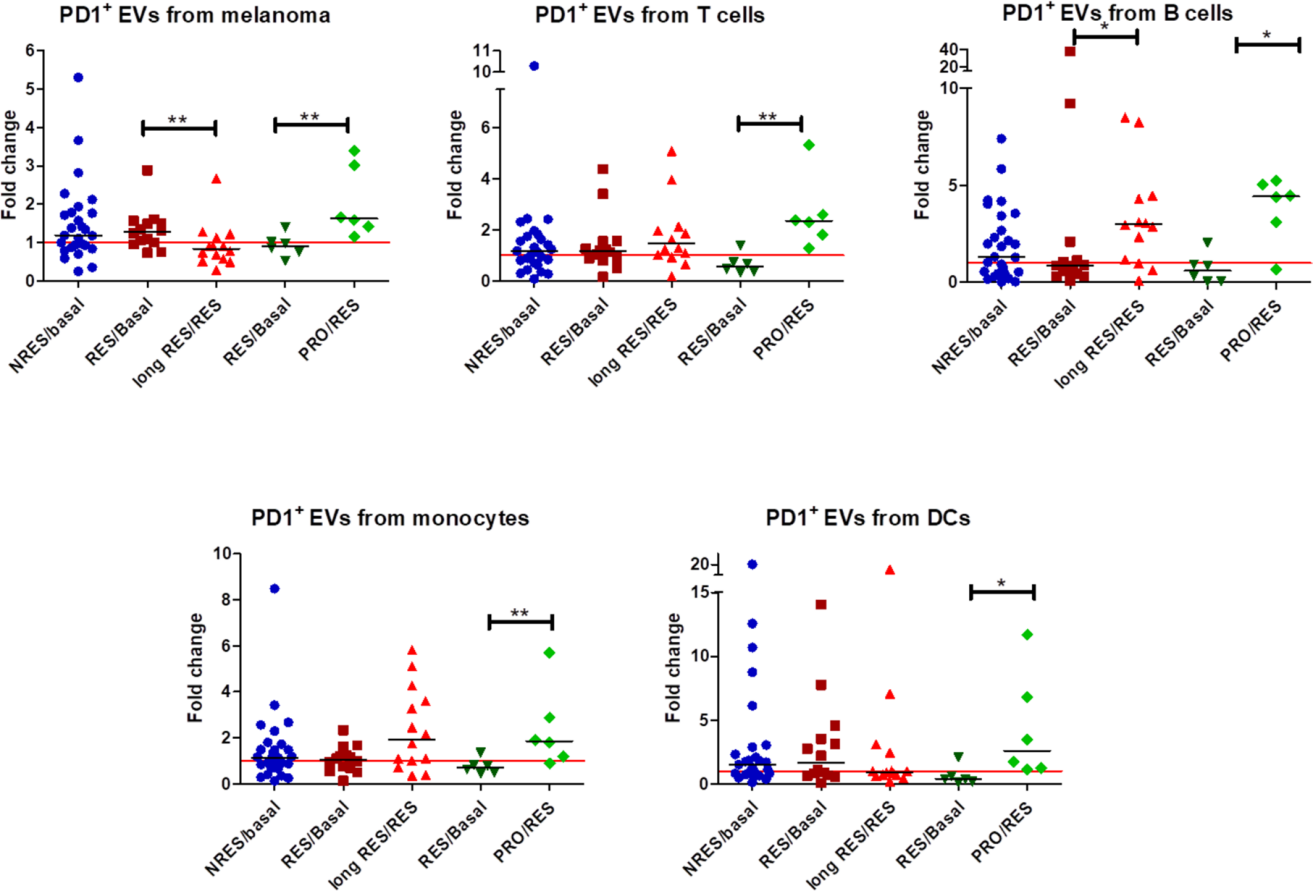
PD1^+^ EVs levels from different cell populations. Scatter plots with median showing PD1.^+^ EVs levels release from melanoma, T cells, B cells, monocytes and DCs as fold change of the first-response/basal and of the second-response/first-response or of the progression/response of NRES, long RES and RES > PRO MM patients. (*p < 0.05, **p < 0.01)

In order to understand whether circulating cell-specific extracellular vesicles may serve as monitoring biomarkers of response to anti-PD1, we performed the quantification of PD1^+^EVs, PD-L1^+^EVs and uPAR^+^EV sub-populations released from tumor cells and immune cells at different time of disease re-evaluation. The results summarised in Fig. 2 showed that only PD1^+^ EVs from melanoma cells were statistically reduced as a function of treatment time in long RES while in RES > PRO, after a slight reduction concomitant with the response, they markedly increased as responders progressed. A similar behaviour was also showed by circulating EVs from DCs but the reduction of these EVs in long RES wasn’t statistically significant. The treatment with anti-PD1 induced a slight increase in PD1^+^ EVs of various origins, with median values ranging from 1.136 to 1.512, when they were isolated from NRES while in all other samples from both long RES and RES > PRO, PD1^+^ EVs increased as a function of time and regardless of clinical response (Fig. 2).

PD-L1^+^ EVs from melanoma and immune cells showed homogeneous behaviour with a slight increase in NRES and a higher increase in responders both in those with long response and in those who progressed (Fig. S3A). Regarding uPAR^+^ EVs, anti-PD1 treatment did not induce statistically different release of these EVs with the exception of those isolated from B cells of RES > PRO which increased with the progression of the disease (Fig. S3A). Therefore, of all these circulating EV subpopulations, only those positive for PD1 and originating from melanoma cells could be used for therapy monitoring, also suggesting that they can actively participate in the acquired resistance to anti-PD1 through a further increase in the seizure of the drug by PD1 positive EVs, as we previously demonstrated [8].

The modifications in the levels of the various subpopulations of circulating EVs of various origins in the plasma of NRES vs long RES vs RES > PRO, led us to investigate whether circulating EVs could induce a change in the metastatic potential of melanoma cells and how these modify the behaviour of immune cells, thus affecting the response to anti-PD1.

### After immunotherapy, do circulating EVs modify the metastatic power of cancer cells?

One of the two BRAF wt cell lines utilised for the evaluation of their metastatic potential in function of the EVs presence was established in our Lab from surgical specimen of a MM patient and named MGS. BRAF wt MGS cells were characterised evaluating the expression of two conventional markers of melanoma, such as PMEL and S100B and as shown in Fig. S3C, all cells were positive for both markers confirming the origin from melanoma.

In order to monitor the metastatic power of melanoma cells after exposure to circulating EVs isolated from i) NRES (before or after anti-PD1 treatment); ii) long RES (pre-therapy and after a prolonged response) and iii) RES > PRO (pre-therapy, when they responded and at progression), we evaluated the ability of all circulating EVs in modulating the invasive behaviour of four MM cell lines, BRAF^V600^ mutant: Hmel-1 (red) and Hmel-9 (black) and BRAF wt: LND-1 (red) and MGS (black).

**Fig. 3:**
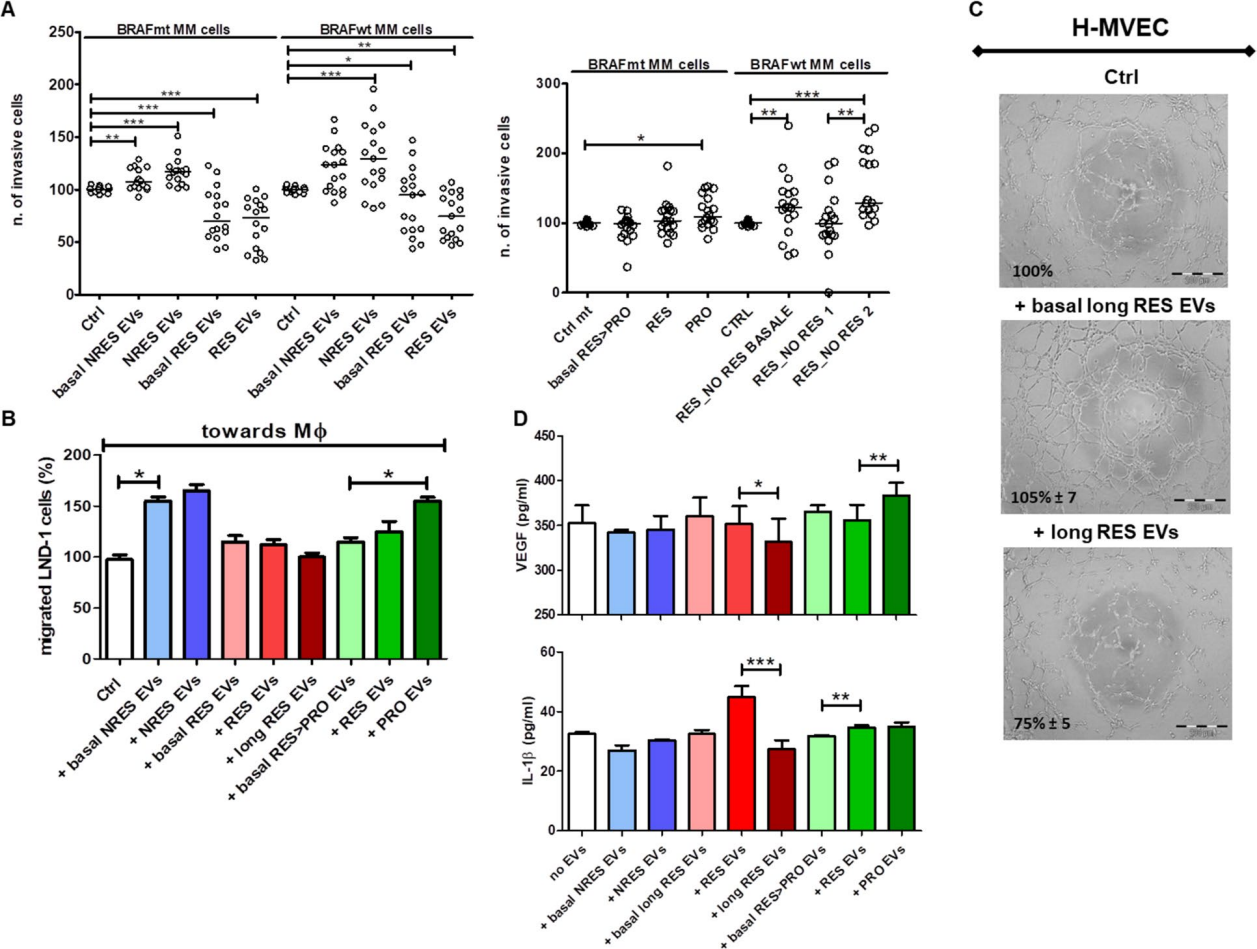
Role of circulating EVs in tumor cell invasion and migration ability and in neoangiogenesis. **A** Scatter plots with median reporting the number of invasive MM cells (BRAF wt or mutated) in presence or absence of circulating EVs from NRES, long RES and RES > PRO (before and after ICI) (**p < 0.01). **B** Bar plot showing the % (mean ± SD) of migrated LND-1 cells, as respect to control without EVs, towards M0 in presence of circulating EVs from NRES, long RES and RES > PRO (before and after ICI). **C** Representative images of H-MVECs in vitro capillary morphogenesis in presence or absence of circulating EVs from long RES (before and after ICI) (scale bar = 200 µm). **D** Bar plots reporting the amount of VEGF and IL-1β release (pg/mL) from M0 in presence or absence of circulating EVs from NRES, long RES and RES > PRO (before and after ICI), quantified by Bioplex assay. (*p < 0.05, **p < 0.01, ***p < 0.001)

The results of the cells invasiveness carried out as described in Methods section are shown in Fig. 3A. Circulating EVs from NRES, before ICI, increased significantly the invasiveness in both BRAF wt and mt cells and the effect was even more amplified with the addition of EVs from NRES after anti-PD1 treatment. Circulating EVs of long RES induced a statistically significant decrease of the invasiveness of all MM cells but after therapy the inhibition of the invasive behaviour seemed to continue only in the BRAFwt cells. These data allowed us to hypothesize that circulating EVs from MM patients influence the metastatic power of MM cells, allowing for an increase of this when originating from NRES. Furthermore, the data suggest that the therapy induced a further increase in invasiveness, although not statistically significant.

The analysis of the three time points of the EVs isolated from RES > PRO evidenced at the progression time the anti-PD1 treatment seemed to increase the invasive characteristics mainly in BRAFwt MM cells (Fig. 3A).

It is known that macrophages participate in the formation of the premetastatic niche responsible for the formation of distant metastases [25]. We therefore investigated whether the presence of circulating EVs modified the migration ability of BRAF wt LND-1 cells, which showed an increased invasive behaviour after ICI, towards the M0 non-activated macrophages that represent the metastatic niche in our simplified cellular model [26]. We found that circulating EVs from NRES strongly increased the migration of LND-1 cells toward M0 as respect those of responders (Fig. 3B). The anti-PD1 slightly increased the ability of tumor cells to migrate when exposed to circulating EVs from NRES while it was reduced when EVs are released from responders (Fig. 3B). Considering the RES > PRO population, we observed that the migration of tumor cells was more stimulated from the circulating EVs isolated from patients who had progressed as respect those isolated from the same patients when responded to ICI, confirming the ability of ICI treated-circulating EVs to increase the metastatic potential of BRAF wt MM cells (Fig. 3B).

In addition, we investigated whether circulating EVs modulated neoangiogenesis. We found that the anti-PD1 modified only circulating EVs released late from long RES which became able to reduce H-MVEC ability to organize in interconnecting tubular structures of about 25% (Fig. 3C). Conversely nor EVs pre-therapy nor those after treatment from the other groups of patients showed to impact the vascular morphogenesis of H-MVECs (Fig. S3C). Angiogenesis is stimulated by vascular endothelial growth factor (VEGF) which induces the openings between the cell junctions and the migration of precursor endothelial cells to form the lining of a new blood vessel [27] and it’s known that macrophages release VEGF when they adapt to the tumor microenvironment [28], hence we wondered if the circulating EVs of MM patients (pre- and post-treatment) could modulate the release of this factor. The analysis of the supernatants of M0 showed a significant and progressive reduction of VEGF released from such cells when they were in presence of circulating EVs isolated from long RES and a significant increase of VEGF, when the M0 were in presence of EVs of RES > PRO patients who progressed after a preliminary positive response (Fig. 3D). The analysis of several cytokines released from M0 after exposure to circulating EVs evidenced that also the release of IL-1β was significantly reduced by the EVs of long RES, while its level increased in the supernatant when M0 were treated with the EVs isolated from RES > PRO patients who still responded and afterwards progressed. Although the level of VEGF and IL-1β showed to be associated only in long responders, we can speculate that the reduction of VEGF was a consequence of the reduction of IL-1β, as suggested by [29].

### What are the possible mechanisms mediated by the circulating EVs that modulate the response to immunotherapy?

**Fig. 4:**
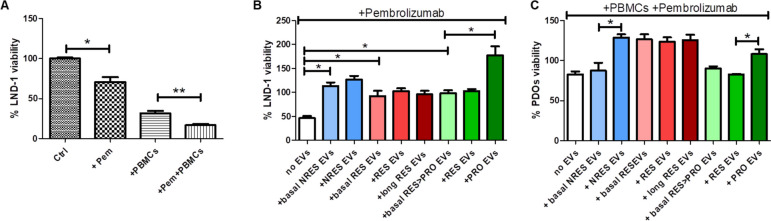
Circulating EVs affect the response to Pembrolizumab in BRAF wt MM cells and responder PDOs **A** Bar plots showing the % of LND-1 cell viability, reported as mean ± SD, treated with 20 µg/mL Pembrolizumab (Pem) for 72 h, with the addition or not of activated PBMCs from a MM responder patient (*p < 0.05, **p < 0.01), **B** in presence of circulating EVs from NRES, long RES and RES > PRO (*p < 0.05). **C** Bar plots showing the viability (%) of MM responder patient PDOs, reported as mean ± SD, treated with 20 µg/mL Pembrolizumab for 72 h, with the addition or not of activated autologous PBMCs, in presence or absence of circulating EVs from NRES, long RES and RES > PRO (before and after ICI)

To determine, in isolated cell models, the ability of circulating EVs to modify the behaviour of immune cells thus elucidating the mechanisms responsible for the clinical response, we initially investigated their ability to impact on the growth of melanoma LND-1 cells (2D model) and MM patient-derived organoids (3D-PDOs) from a RES, when exposed to anti-PD1 in presence of PBMCs isolated from the same patient and activated as described in Methods section. To perform the cytotoxicity study of Pembrolizumab, we preliminary tested the 1:1 and 1:5 ratio of tumor cells:PMBCs, and as the 1:1 ratio achieved almost 15% inhibition of cell viability, in agreement with our previous data [8], in order to amplify the impact of EVs on the response to anti-PD1 we chose to conduct the assay by using the 1:5 ratio of tumor cells:PMBCs. Regarding the concentration of EVs, we used 10 ^7^ EVs for the experiments with LND-1 cell line and 10 ^8^ EVs for the experiments with organoids because per se higher concentrations strongly inhibited the viability of LND-1 cell line and did not allow for discriminating the impact of EVs on the resistance to anti-PD1.The LND-1 cells treated with Pembrolizumab showed a reduction of cell viability of 30% and it increased to 50% in the presence of activated PBMC, suggesting that the removing of the immune system block, due to the interaction between PD1 and PD-L1, increased the immune-mediated cytotoxicity (Fig. 4A). The addition of circulating EVs, isolated from pre-treatment plasma of NRES, completely abolished the response of LND-1 to Pembrolizumab/PBMCs and indeed induced stimulation in the proliferation of cancer cells which further increased when we added EVs coming from the ICI-treated NRES (Fig. 4B). Conversely, circulating EVs from RES, even if completely abolished the response to Pembrolizumab/PBMCs, did not appear to further affect cell proliferation (Fig. 4B). In the RES > PRO model circulating EVs, before therapy and when the patient responded to therapy, abolished the response to anti-PD1 and, in the presence of EVs isolated after progression, a statistically significant increase in proliferation was observed (Fig. 4C). Thus, it should be emphasized that circulating EVs induced an increase in proliferation when coming from samples of patients who did not respond to anti-PD1 both in the first and second instances. The lack of response of the LND-1 cells to anti-PD1/PBMCs when the experiment was conducted in the presence of circulating EVs could depend on the absence of the tumor microenvironment which contributes to the immunotherapy response [30].

Consequently, we decided to carry out the same experiments using an ICI responsive model, the PDOs of a responder containing the tumor microenvironment in addition to melanoma cells. We observed a reduced response to Pembrolizumab/PBMCs in the absence of circulating EVs (about 80%) but differently from what we observed in the 2D model, the EVs from NRES did not seem to affect the response while, if they were obtained from NRES after ICI, they reduced the treatment response in a statistically significant way (Fig. 4C). Conversely, pre-therapy RES-EVs reduced the effect of pembrolizumab and this effect was not dependent on the anti-PD1 treatment (Fig. 4C). The circulating EVs of RES > PRO showed the same behaviour than in the 2D cell model (Fig. 4C). Thus, we demonstrated that the EVs from NRES, long RES and RES > PRO modulated in various ways the proliferation of melanoma cells in both 2D and 3D models, in agreement with the response to anti-PD1.

Therefore, we tried to investigate how these EVs modulated the activity of immune cells, responsible for the clinical response to anti-PD1, focusing on T cells, DCs and macrophages.

**Fig. 5:**
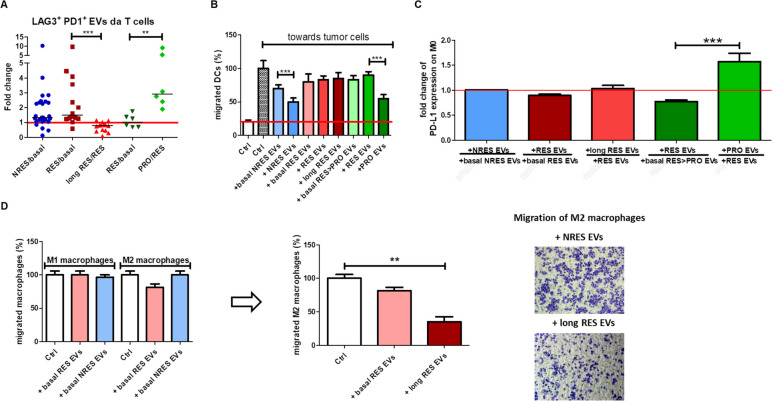
Circulating EVs are involved in the response to immunotherapy by affecting immune cells behaviour. **A** Scatter plot with median showing the amount of LAG-3^+^PD1^+^ EVs from T cells, reported as the fold change of the first-response/basal and of the second-response/first-response or of the progression/response of NRES, long RES and RES > PRO MM patients (***p < 0.001, **p < 0.01). **B** Bar plot reporting the mean ± SD of migrated DCs (%) towards LND-1 cells, in presence or absence of circulating EVs from NRES, long RES and RES > PRO MM patients (before and after ICI). **C.** Bar plot showing PD-L1 expression in M0 macrophages, as a fold change of PD-L1^+^ M0 macrophages co-incubated with NRES, long RES and RES > PRO circulating EVs (first-response/basal, second-response/first-response or progression/response). **D** Bar plots representing the percentage of migrated M1-M2 macrophages in presence or absence of circulating EVs from NRES, long RES and RES > PRO MM patients (before ICI, and long RES before and after ICI) (**p < 0.01); representative images of transwell migration assay of M2 macrophages co-incubated with NRES and RES (best response) circulating EVs

The response to anti-PD1 depends on the functionality of the T cells; it has been shown that when they co-express high levels of PD1 and LAG3 compared with T cells expressing only LAG3 or PD1, the T cells are exhausted [31]. We verified, as indirect evidence of the increase in PD1^+^ LAG3^+^ T cells, if the circulating EVs positive for both PD1 and LAG3 and released by CD8 + T cells increased after immunotherapy (Fig. 5A). The median value of the ratios between the percentage of PD1^+^LAG3^+^ EVs, isolated from T cells, after and before anti-PD1 treatment in NRES was 1.33 evidencing that ICI increased the release of this subpopulation of EVs; this increase was evident at the beginning of the response in long RES but with the prolongation of the treatment the release was drastically reduced (1.5 *vs* 0.7804), indirectly indicating that in the prolonged responses to anti-PD1 there is a reduction in exhausted T cells. As expected, the situation was reversed in RES > PRO; after a lower increase in the double positive EVs when the patients responded, a significant increase in the same EV population was observed when the patients progressed (median values: 1.030 *vs* 2.920), thus, we hypothesise the increase in exhausted T cells.

A reduction of intratumoral DCs could be a reason for anti-PD1 failure as well as the lack of tumor-infiltrating T cells or high level of exhausted T cells [32]. Thus, the recruitment of DCs in TME could be considered another relevant step in determining the response to anti-PD1. In order to evaluate if circulating EVs are able to modify the migration of DCs cells toward melanoma, we evaluated the directed movement of DCs in response to EVs isolated from the same three subsets of patients (Fig. 5B). As known, tumor cells release signals which accelerate the migration of the DCs, and in our experimental condition the increase was of about 5 folds. The addition of circulating EVs from NRES and RES reduced instead the activation of 30% and 20%, respectively. In NRES treated with anti-PD1, circulating EVs reduced the ability of DCs to reach the tumor while in RES this was maintained. Situation that became dramatically evident in patients who after the initial response to immunotherapy progressed by releasing EVs that strongly inhibited the migratory capacity of DCs.

Another population of immune cells strongly involved in the response to anti-PD1 are macrophages. The role of circulating EVs in the functionality of macrophages was investigated analysing their ability i) to modulate the expression of PD-L1 in macrophages, ii) to release selected cytokines involved in the establishment of the immunosuppressive phenotype and in the development of the neoangiogenesis and iii) to modulate the migration of the two subtypes M1 and M2 towards the tumor.

In the literature it’s reported that circulating EVs from tumor cells increase the expression of PD-L1 in macrophages giving them an immunosuppressive phenotype with a subsequent reduction of the immune response by inhibiting the functions of effector T cells [33]. We focused on the determination of PD-L1^+^ macrophages after exposure to circulating EVs from NRES, long RES and RES > PRO as an index of the polarization of these immune cells toward the immunosuppressive "non-classical M1" phenotype depending from the expression of PD-L1 and cytokines release. Our FCM results demonstrated that the expression of PD-L1 in M0 was not modulated by the exposure to circulating EVs from NRES treated with ICI (fold change = 1) while we observed a slight, early but transient reduction of PD-L1^+^ M0 after the addition of long RES EVs (Fig. 5C and S4). RES > PRO circulating EVs reduced the percentage of PD-L1^+^ M0 at the beginning of therapy but, when the patient progressed, the EVs induced a significant increase of them (Fig. 5C and S4).

Several cytokines are involved in determining the immunosuppressive phenotype of macrophages [29, 34–37] and we analysed how circulating EVs modified the release of a panel of 27 cytokine and chemokine cell signaling molecules from M0; among them, we focused on G-CSF, GM-CSF, IFN-γ, IL1b, IL-1ra, IL-6, IL-8, IL-10, IL-12, IP-10, MCP-1, RANTES and VEGF because reported as positive or negative mediator of the immunosuppressive phenotype and of angiogenesis. Our results showed that the release of G-CSF, GM-CSF, IFN-γ, IL-6, IL-8, IL-10 and IL-12 were not modulated by circulating EVs (data not shown). IL-1β and IL-1ra are released by macrophages and have an immunosuppressive role by stimulating the myeloid-derived suppressor cells (MDSCs) and M2-polarized macrophages [34, 38]. The addition of circulating EVs of various origin to M0 macrophages induced, when they came from ICI-treated patients, a tendency to increase the level of IL-1β and IL-1ra, which showed a drastic reduction only in long responders (Fig. 3D and Fig. S5). Two chemokines whose release is modulated by the addition of the circulating EVs to the M0 macrophages are MCP-1 and RANTES. Macrophages are among the major producers of the two chemokines that regulate the migration and infiltration of various immune cells, such as the macrophages themselves, memory T cells and natural killer (NK) cells [39, 40]. The release of MCP-1 and RANTES from M0 after the addition of circulating EVs of various origins was not strongly modulated; however a slight reduction of their levels were evident in the long RES (Fig. S5).

Furthermore, we analysed if the circulating EVs of NRES and RES affected macrophages M1 and M2 migration. Pre-therapy EVs didn’t modulate their migration with the exception of those from RES which selectively affected M2 migration (about 20%); the EVs from RES after ICI induced a dramatic reduction of M2 migration (Fig. 5D). Results are in agreement with the increase of the immunosuppression in NRES and with the attempt of macrophage to reactivate the immune system when the therapy is effective as also evidenced by the reduced ability of M2-like to migrate to the tumor in long RES.

## Discussion

The lack of response at the beginning of ICI or the onset of drug resistance are serious troubles, first of all for the MM patients in whom the disease progresses and suffer from side effects of the ICI, for clinicians who do not have valid therapeutic alternatives and for the National Health Institute for the high cost of the immunotherapeutic drugs. Therefore, identifying valid biomarkers for the selection of patients to be treated or for the monitoring of the response to immunotherapy is an urgent need.

Liquid biopsy, with its high compliance for the patient, is an excellent starting point for the analysis of biomarkers and if the dosage of them was possible directly in the blood, plasma or serum without further laboratory procedures, these biomarkers would be extremely promising. Pursuing this hypothesis, we wanted to evaluate whether the soluble immune checkpoints, main actors of immunotherapy with anti-PD1, PD1 and PD-L1, could be used for the selection of patients to be treated with ICI or for monitoring of the response to therapy over time.

Unfortunately, in our experience neither the characterization conducted on a large number of plasma samples obtained from patients before the start of therapy nor that conducted on post-treatment blood samples (longitudinal study) have shown that sPD1 and sPD-L1 are valid biomarkers. Our results are in agreement with literature evidences reporting both a dramatic increase of sPD1 in plasma upon anti-PD1 treatment, regardless of response to therapy, and the absence of its predictive value for the response to this immunotherapy approach when measured in serum of MM patients [10, 41, 42]. Therefore, we confirm that they could not be reliable biomarkers for the selection of MM patients to treat with anti-PD1 and for the monitoring of ICI response.

Some authors such as Machiraju and co-authors suggested that the increased presence of sPD1 in the blood may directly limit the efficacy of the therapeutic anti-PD1 antibody by competing with its binding to membrane-bound PD1 on immune cells and that increased sPD1 concentrations in melanoma patients indicated resistance to ipilimumab plus nivolumab combined treatment but not to anti-PD1 monotherapy [41]. Our study together with the previous ones [7, 8] allowed a breakthrough in the characterization of the role of circulating PD1 and PD-L1, having shown that only a part of them, i.e. three subpopulations of circulating EVs positive for the two checkpoints and released from melanoma cells, T cells and DCs, was actively involved in the prediction of response/resistance to anti-PD1 and closely related to the innate anti-PD1 resistance [8].

Since liquid biopsy also offers clinicians the possibility to follow cancer patients during their treatments, in this study we evaluated if any subpopulation of circulating EVs could be used for the monitoring of resistance to anti-PD1.

For the first time, we demonstrated that among the circulating EVs positive for PD1, PD-L1 or uPAR released by melanoma cells or immune cells such as T and B cells, monocytes and DCs, only the percentage of those positive for PD1 and released by melanoma cells was reduced in long RES in function of time, while increased in RES when progressed. Therefore, we propose the melanoma-derived PD1^+^EVs as new promising biomarker to track the response to anti-PD1 in real time and predict the early development of resistance to this immunotherapy approach allowing treatment adjustment. The idea of using circulating EVs in the monitoring of therapy responses has been already investigated in cancer diseases such as prostate cancer, HNSCC, pancreatic cancer, colorectal cancer and breast cancer [43, 44]. In melanoma, it has been explored in 2018 by Costa Svedman demonstrating that some miRNAs transported by circulating EVs increased during treatment with MAPKis in BRAF mutated MM patients [45]. More recently, Wang and co-authors demonstrated the feasibility of monitoring patient responses to targeted therapy with BRAFi ± MEKi determining the plasma EV phenotypic evolution using a multiplex approach (MCSP, MCAM, ErbB3 and LNGFR) even if their results are not conclusive for the identification of biomarkers for therapy monitoring [44].

Furthermore, the strong increase in PD1^+^ EVs from melanoma cells, in both NRES and RES when progressed, could be responsible for a greater sequestration of anti-PD1 in the bloodstream [8], suggesting a close relationship between these EVs and the establishment of acquire resistance to anti-PD1.

Other investigated topic was the exploration of the therapy resistance mechanisms dependent from circulating EVs through the increased metastatic potential of melanoma cells and the immune-suppression of the T cells, DCs and macrophages.

We showed that ICI strengthens the ability of circulating EVs to impact on the metastatic potential of melanoma cells mainly in BRAF wt ones, increasing their invasive and migration ability towards the metastatic niche, in the formation of which macrophages play a key role [25]. Although, they had no impact on neoangiogenesis, which is reduced only after the addition of EVs from long RES, probably trough the reduction of the release of VEGF and IL-1β, the involvement of which has already been reported [28, 29]. The role of EVs in the formation of premetastatic niches has been already hypothesised in melanoma and in breast cancer and correlated with the formation of cancer-associated fibroblasts (CAFs) [46, 47] and not of macrophages, as we showed. The correlation between EVs and the pre-metastatic niches is reported also in other cancer diseases, such as in lung cancer, through the recruitment of neutrophil mediated by tumor exosome [48], and in pancreatic cancer, in which the bone-marrow macrophages are recruited for the formation of the early stages of pre-metastatic niche mediated by the exosome in the liver [47–49].

Circulating EVs have been suggested to be mediators utilised by cancer cells to promote immune escape, by transporting PD1 and PD-L1 and changing the phenotypic characteristics of different immune cell populations such as T cells, DCs and macrophages [8, 31–34, 38–40, 47, 50, 51]. In our study, for the first time, we focused on how anti-PD1 modifies the circulating EVs and their ability to affect the immune system, suggesting that in MM patients with innate resistance to anti-PD1, the exposure to ICI induced a reduction of the functionality of the T cells which became exhausted, as suggested by the increase of CD8^+^LAG3^+^PD1^+^ EVs, and by the reduction of the recruitment of DCs in TME. In long RES, long-term therapy resulted in reduced release of CD8^+^LAG3^+^PD1^+^ EVs suggesting a lesser induction of exhaustion in T cells and reduced migration of M2 macrophages. The opposite effect was evident when EVs were released from patients who progressed after an initial response to therapy, since they induced a further increase of immunosuppressive "non-classical M1" macrophages.

## Conclusions

Herein, for the first time we have identified a subpopulation of circulating PD1^+^ EVs coming from melanoma cells, as promising biomarkers for the monitoring of anti-PD1 response. Furthermore, we provided evidences that circulating EVs are responsible for the establishment of acquired resistance to anti-PD1, the increased metastatic potential of melanoma cells and the immune-suppression of the T cells, DCs and macrophages. Thus, we provided the rational for using this “selected” subpopulation of circulating EVs for monitoring the response to ICI in MM patients with the great advantage of dosing them in liquid biopsy, so a minimally invasive procedure. An open question in the EV studies is their determination directly in the plasma. For this reason, we plan to design and implement a microfluidic system for a rapid and cheap determination of PD1^+^ EVs that could allow clinicians to monitor the response to anti-PD1 with great advantages both for patients and for the National Health System.

### Supplementary Information

**Additional file 1:** **Fig. S1. **sPD1 and sPD-L1 levels in plasma of MM patients underwent immunotherapy with ICI. **Fig. S2.** MM patients circulating EVs characterization. **Fig. S3.** Effect of anti-PD1 treatment on PD-L1^+^ or uPAR^+^ EVs release and neoangiogenesis. **Fig. S4. **Influence of circulating EVs on PD-L1 expression in M0 macrophages. **Fig. S5.** Modulation of cytokines and chemokines released from M0 macrophages after treatment with circulating EVs of MM patients.

## Data Availability

All data and material are available at the Laboratory of Experimental Pharmacology of IRCCS Istituto Tumori Giovanni Paolo II. The data regarding the EV characterization presented in this study are openly available in the EV-TRACK knowledgebase [52]. [EV-TRACK ID: EV220411].

## Abbreviations

CR: Complete response
CTLA-4: Cytotoxic T-lymphocyte-associated protein 4
DCR: Disease control rate
DCs: Dendritic cells
DMEM: Dulbecco's modified Eagle's medium
EVs: Extracellular vesicles
FBS: Fetal bovine serum
FCM: Flow cytometry
ICI: Immune checkpoint inhibitors
IF: Immunofluorescence
MM: Metastatic melanoma
H-MVECs: Human Dermal Microvascular Endothelial Cells
NRES: Non-responder
NTA: Nanoparticle tracking analysis
ORR: Overall response rate
OS: Overall survival
PDOs: Patient-derived organoids
PFS: Progression free survival
PR: Partial response
RES: Responder
SD: Stable disease
sPD1: Soluble forms of PD1
sPD-L1: Soluble forms of PD-L1
T cells: T lymphocytes
THP-1: Human monocytic leukemia cell line
uPAR: Urokinase-type Plasminogen Activator Receptor
VEGF: Vascular Endothelial Growth Factor
