# Supplementary material for: Circulating extracellular vesicles are monitoring biomarkers of anti-PD1 response and enhancer of tumor progression and immunosuppression in metastatic melanoma
Source: J Exp Clin Cancer Res. 2023 Sep 28;42:251. doi: 10.1186/s13046-023-02808-9 (PMC10538246; doi:10.1186/s13046-023-02808-9)
Supplement: Supplementary file 1 — Additional file 1: Fig. S1. sPD1 and sPD-L1 levels in plasma of MM patients underwent immunotherapy with ICI. Fig. S2. MM patients circulating EVs characterization. Fig. S3. Effect of anti-PD1 treatment on PD-L1+ or uPAR+ EVs release and neoangiogenesis. Fig. S4. Influence of circulating EVs on PD-L1 expression in M0 macrophages. Fig. S5. Modulation of cytokines and chemokines released from M0 macrophages after treatment with circulating EVs of MM patients. [file 13046_2023_2808_MOESM1_ESM.docx]

**Supplementary informations**

**Supplementary Figures legend**

**Fig. S1. sPD1 and sPD-L1 levels in plasma of MM patients underwent immunotherapy with ICI.** Scatter plots with median showing sPD1 and sPD-L1 plasma levels (pg/mL) of NRES, long RES and RES>PRO MM patients (before and after ICI), measured by ELISA assay. (***p<0.001, **p<0.01).

**Fig. S2. MM patients circulating EVs characterization. A.** Representative NTA histogram with confidence interval reporting the concentration and specific particle size of circulating EVs. **B.** Dot plots of FCM analysis showing the double expression of CD9/CD63, CD9/CD81 and CD63/CD81 in circulating EVs. **C.** Violin plot of FCM analysis results reporting the percentage of circulating EVs from melanoma cells, T cells, B cells, monocytes and DCs and scatter plots with median of the results of the same analysis showing the percentage of circulating EVs from NRES, long RES and RES>PRO, clustered by cells of origin. **D.** Scatter plots of the same FCM analysis reported as fold change of circulating EVs at first-response/basal and second-response/first-response or progression/response from NRES, long RES and RES>PRO MM patients, clustered by cells of origin. (*p<0.05).

**Fig. S3. Effect of anti-PD1 treatment on PD-L1^+^ or uPAR^+^ EVs release and neoangiogenesis. A.** Scatter plots showing FCM analysis results, reported as the fold change of the first response/basal and of the second response/first response, and of the progression/response of PD-L1^+^ or uPAR^+^ EVs from NRES, long RES and RES>PRO MM patients and from different cells of origin (**p<0.01, *p<0.05). **B.** Representative images of *in vitro* vascular morphogenesis of N-MVECs in presence of circulating EVs from NRES, long RES and RES>PRO MM patients (before and after ICI) (scale bar = 200 µm). **C.** Representative IF images showing the expression of PMEL and S100 in MGS cells; blue = nuclei (DAPI), red = PMEL, green = S100; scale bar = 100µm).

**Fig. S4. Influence of circulating EVs on PD-L1 expression in M0 macrophages.** Dot plots of FCM analysis showing the CD68^+^PD-L1^+^ macrophages population (%) after co-incubation with circulating EVs from NRES, long RES and RES>PRO MM patients (before and after ICI).

**Fig. S5. Modulation of cytokines and chemokines released from M0 macrophages after treatment with circulating EVs of MM patients.** Bar plots reporting the amount of MCP-1, RANTES and IL-1ra (pg/mL) released from M0 macrophages in presence or absence of circulating EVs from NRES, long RES and RES>PRO MM patients (before and after ICI), quantified by Bioplex assay.

**Fig. S1**

**Fig. S2**

**Fig. S3**

**Fig. S4**

**Fig. S5**
